# Neuronal NMDAR Currents of the Hippocampus and Learning Performance in Autoimmune Anti-NMDAR Encephalitis and Involvement of TNF-α and IL-6

**DOI:** 10.3389/fneur.2019.00684

**Published:** 2019-06-26

**Authors:** Xu Wang, Chi Ma, Cai-Yun Liu, Guang-Jian Li, Ding Zhao, Dong-Feng Han

**Affiliations:** ^1^Department of Neurology and Neuroscience Center, First Hospital of Jilin University, Changchun, China; ^2^Department of Neurosurgery, First Hospital of Jilin University, Changchun, China; ^3^Department of Orthopedics, First Hospital of Jilin University, Changchun, China; ^4^Department of Emergency, First Hospital of Jilin University, Changchun, China

**Keywords:** autoimmune disease, encephalitis, neuroinflammation, cytokines, memory deficits

## Abstract

Among autoimmune encephalitis, patients with anti-N-methyl D- aspartate receptor (NMDAR) encephalitis typically present epileptic seizures, memory deficits and psychiatric symptoms. However, the signal mechanisms leading to the functional disorders of autoantibodies are largely unclear. In this study, anti-NMDAR antibody was administered into dentate gyri against the NR1 subunit of the NMDAR. The purpose of the study examined the effects of pro-inflammatory tumor necrosis factor-α (TNF-α) and interleukin-6 (IL-6) on neuronal NMDAR currents of the hippocampus in rats with anti-NMDAR encephalitis and we further determined the role played by TNF-α and IL-6 in modulating learning performance. In results, we observed a decrease in amplitude of the NMDAR-mediated excitatory postsynaptic currents (NMDAR-EPSCs) in the hippocampal neurons of animals treated with anti-NMDAR. In those rats with anti-NMDAR, we also observed impaired learning performance in the Morris water maze and spatial working memory test. Of note, cerebral infusion of TNF-α and IL-6 worsened NMDAR-EPSCs and this was accompanied with exaggeration of impaired learning performance. In conclusion, our findings suggest that the role played by neuroinflammation in exacerbating the memory impairment found in animals treated with anti-NMDAR. Anti-inflammation is a potential target in improving the memory impairment induced by anti-NMDA encephalitis.

## Introduction

In patients with several identified paraneoplastic autoimmune encephalitis, autoantibodies are identified which are against cell surface and synaptic proteins (1, 2). In one of autoimmune encephalitis antibodies against the N-methyl-D-aspartate receptor (NMDAR, a glutamatergic receptor) are found (3, 4). It is noted that numerous functions including learning and memory, cognition and behavior are depend on synaptic plasticity regulated centrally by glutamatergic transmission (5, 6). Thus, in association with the role of NMDARs in glutamatergic transmission and activity-dependent plasticity, in anti-NMDAR encephalitis sudden behavioral, memory, and personality changes are observed and these symptoms can progress to seizures, autonomic instability, and psychiatric symptoms. Irretrievable symptoms and death can occur without treatment; whereas a full recovery for about 80% of patients was reported after appropriate immunotherapy (7). Nonetheless, the mechanisms leading to the functional consequences of such autoantibodies in anti-NMDAR encephalitis are poorly understood.

In particular, defects in glutamate transmission are related to neuropsychiatric disorders, and NMDAR hypofunction is thought as a part of the pathophysiological mechanisms leading to schizophrenia (8). In human and rodent studies, sub-anesthetic doses of NMDAR inhibitors (i.e., phencyclidine and ketamine) have been reported to be psychotomimetic and they cause the stereotypic movements, autonomic instability, and seizures, all of which are characteristic of anti NMDAR encephalitis (9, 10). Pharmacological blockade or genetic knock-down of NMDARs can also alter learning performance, memory function, excitatory-inhibitory balance, and neurological behavior (11–13). Accordingly, it is important to study the consequences of NMDAR hypofunction and the mechanisms of antibody-mediated dysfunction in this disease to better understand pathophysiology of patient symptoms.

In addition, using cultured rats' hippocampal neurons pathophysiological role of anti-NMDAR antibodies was examined. Cerebrospinal fluid (CSF) obtained from anti-NMDAR encephalitis patients was applied to these neuronal cells and this produced a substantial and reversible loss of postsynaptic NMDARs leading to impaired NMDAR-mediated miniature excitatory postsynaptic currents (mEPSCs) after its short-term treatment (14). Also, a long-term potentiation (LTP) determining a classical NMDAR-dependent function was attenuated in mouse hippocampal slices bathed in CSF from anti-NMDAR encephalitis patients (15). These findings suggested that anti-NMDAR is likely to exert NMDAR inhibiting effects. Thus, in this study, we employed electrophysiological methods to examine the activity of mEPSCs in the hippocampal neurons of rats treated with anti-NMDAR.

Of note, a prior study provided evidence for an essential role played by anti-NMDAR antibodies *in vivo*, by demonstrating that anti-NMDAR plays a pathophysiologically relevant role *in vivo* (16). For example, in this prior study, CSF containing anti-NMDAR was stereotactically injected into the rat hippocampus. Substantial deficits in NMDAR-mediated synaptic transmission and plasticity were observed later *in vitro* after *in vivo* application of anti-NMDAR. In addition, in this prior study, Morris water maze experiments showed impairments in learning behavior associated with the hippocampus in the rats injected with anti-NMDAR. It is noted that pro-inflammatory cytokines (PICs) are elevated in the plasma and CSF in the patients with anti-NMDAR encephalitis and neuroinflammation has been reported to contribute to the severity of symptoms presented in patients with anti-NMDAR encephalitis (17–20). Since there is a close relation in neuroinflammation and anti-NMDAR encephalitis, PICs/chemokines have been suggested as biomarkers of this disease and potential therapeutic targets in encephalitis (19, 20). On the basis of those previous findings representative cytokines TNF-α and IL-6 were selected in this report. In this study anti-NMDAR antibody was administered into dentate gyri against the NR1 subunit of the NMDAR and we also examined the protein expression of NR1 in the hippocampus of control rats and rats treated with anti-NMDAR. We hypothesized that a chronic cerebral infusion of TNF-α and IL-6 worsens mEPSCs in the hippocampal neurons of rats treated with anti-NMDAR and this thereby amplifies impairment of learning performance.

## Materials and Methods

### Animals

The guidelines of the International Association for the Study of Pain were followed for all animal protocols which were approved by the Institutional Animal Care and Use Committee of Jilin University. Adult male Sprague-Dawley rats weighting 200–250 g were housed in a temperature-controlled room (25°C) on a 12/12 h light/dark cycle and they had free access to food and water.

### Antibody Injection

After the rats were anesthetized with sodium pentobarbital (45 mg/kg, i.p.), they were mounted on a stereotaxic frame (Stoelting Co.). A midline incision was made to expose the skull and one burr hole was drilled. Bilateral stereotaxic injection was performed. The injection of 5 μl of anti-NMDAR1 (50 ng/μl, dissolved in CSF; Merck Millipore, Billerica, MA, USA) into dentate gyri [coordinates: 5.2 mm posterior, ±4.3 mm lateral, 4.8 mm deep (relative to bregma)] was performed at each side with a Hamilton syringe connected to a syringe pump. In control rats, 5 μl of CSF was injected in the similar way. The injection was performed at a rate of 0.25 μl/min (over 20 min) via a perfusion pump. One to seven days following the injection learning behavior experiments and electrophysiological experiments were performed accordingly.

In a subset of animals, histological examinations were performed to examine the localization of the stereotactic injection into the dentate gyrus. In this procedure, 0.5 μl of 2% Evans blue was given through the dentate gyrus. Then, the animals were anesthetized with sodium pentobarbital and intra-cardiacally perfused with physiological saline followed by 4% of paraformaldehyde solution. The hippocampus was sectioned and the location of injection sites was verified by identification of blue dye according to the atlas of Swanson (21).

### Administration of Drugs

After completion of antibody injection, drugs were given. The following procedures were performed as described in our previous publication (22). Animals were cannulated with an L-shaped stainless steel cannula aimed at the lateral ventricle (coordinates: 3.7 mm posterior to the bregma, 4.1 mm lateral to the midline, and 3.5 mm under the dura). The guide cannula was fixed to the skull using dental zinc cement and jewelers' screw. Then, the cannula was connected to an osmotic minipump (Alzet pump brain infusion kit, DURECT Inc., Cupertino, CA) with polycarbonate tubing. The pumps were placed subcutaneously between the scapulae, and loaded with vehicle (CSF) as control or TNF-α (5 μg) and IL-6 (5 μg), respectively. Those agents were delivered for a period of 7 days (a rate at 0.25 μl/h). This intervention allowed animals to receive continuous intracerebroventricular (i.c.v.) infusion via the osmotic minipumps. After this procedure, animals were kept in individual cages to secure cannulation and brain infusion kit.

### Western Blot Analysis

The tissues from individual rats were sampled for the analysis as described in our prior work (22). In brief, the hippocampus of the rats was removed. Total protein was then extracted by homogenizing the sample in ice-cold immunoprecipitation assay buffer with protease inhibitor cocktail kit (Promega Co. Madison, WI, US). The lysates were centrifuged and the supernatants were collected for measurements of protein concentrations using a bicinchoninic acid assay reagent kit. After being denatured, the supernatant samples containing 20 μg of protein were loaded onto gels and electrically transferred to a polyvinylidene fluoride membrane. The membrane was incubated overnight with primary antibodies (diluted at 1:500): rabbit anti-NR1 and anti-GluR2/3. The membranes were washed and incubated with an alkaline phosphatase conjugated anti-rabbit secondary antibody (1:1000). The primary and secondary antibodies were obtained from Abcam Co. or Antibodies online Com. Enhanced chemiluminescence was used to detect the immunoreactive proteins and the primary antibody recognized on the bands was visualized by exposure of the membrane onto an x-ray film. To show equal loading of the protein the membrane was stripped and incubated with anti-β-actin. After the film was scanned, the optical density of NR1/GluR2/3/β-actin bands was analyzed using the Scion Image software.

### Electrophysiological Experiments

The rats were anesthetized with sodium pentobarbital (75 mg/kg, i.p.) and decapitated. Briefly, the brain was taken out and placed in ice-cold artificial cerebral spinal fluid (aCSF) solution. The aCSF perfusion solution contained 124.0 NaCl, 3.0 KCl, 1.3 MgSO_4_, 2.4 CaCl_2_, 1.4 NaH_2_ PO_4_, 10.0 glucose, and 26.0 NaHCO_3_ (in mM). A tissue block of the hippocampus was glued onto the stage of the vibratome and coronal slices (300 μm) were cut from the tissue block in ice-cold aCSF solution. Sixty minutes was allowed to incubate the slices in the aCSF at 34°C, saturated with 95% O_2_-5% CO_2_ before being transferred to the recording chamber.

A whole cell voltage-clamp mode was employed to record postsynaptic currents of hippocampal neurons. Borosilicate glass capillaries (1.2 mm OD, 0.69 mm ID) were pulled to make the recording pipettes. The resistance of the pipette was 4–6 MΩ as it was filled with the internal solution [contained 130.0 potassium gluconate, 1.0 MgCl_2_, 10.0 HEPES, 10.0 EGTA, 1.0 CaCl_2_, and 4.0 ATP-Mg (in mM) with pH 7.25 and osmolarity of 280–300 mOsm]. The slice was placed in a recording chamber perfused (at 3.0 ml/min) with the aCSF (containing 0 Mg^2+^) saturated with 95% O_2_-5% CO_2_. An in-line solution heater was used to keep the temperature of the perfusion solution at 34°C. Whole cell recordings from hippocampal neurons were performed visually using differential interference contrast (DIC) optics on an upright microscope (BX50WI, Olympus) and a tight giga-ohm seal was subsequently obtained in hippocampal neuron viewed using DIC optics (23). A MultiClamp 700B amplifier digitized with a DigiData 1440A was used record signals were recorded. A liquid junction potential of −15.0 mV was corrected during off-line analysis (23) and 15 min was allowed after the recording reached a steady state.

At a holding potential of −70 mV, the mEPSCs were obtained in the presence of TTX (1 μM) and picrotoxin (10 μM). 2-amino-5-phosphonopentanoic acid (APV, a NMDA receptor antagonist in 50 μM) and 6-cyano-7-nitroquinoxaline-2,3-dione (CNQX, AMPA receptor antagonist in 20 μM) were used to block NMDAR- and AMPAR-mediated currents, respectively. Detection of events was accomplished by setting a threshold above the noise level (23) and the mEPSCs of the hippocampal neurons were analyzed off-line with a peak detection program (MiniAnalysis, Synaptosoft, Leonia, NJ).

### Learning Behavior Experiments

The hidden platform task in the Morris water maze was used to examine learning behavior. The circular pool was consisted of polypropylene (diameter: 150 cm; water depth: 50 cm; and platform diameter: 7 cm). The pool was filled with opaque water and maintained at 21 ± 1°C. Four large black-and- white cues (a black cross on 10 cm of white square cardboard) were located at four different sites (east, south, west, and north) and 25 cm above the platform. On the day before the experiments, all rats were required to explore the water maze without platform for acclimatization. The platform was inserted below the water level (1–2 cm) and each rat was randomly assigned to one of four different platform locations on the first day. Then, each rat was given six consecutive trials to reach the platform from days 1 to 7. The starting points were chosen in a random fashion (six out of eight different positions). If a rat failed to reach the platform within 60 s, it was placed on the platform. In any case, a rat was allowed to stay on the platform for another 30 s, before it was moved back into the cage. The next trial was then started following a recovery time of 60 s in the cage. To analyze swimming path length and swimming speed the Ethovision Color software (Noldus Beijing, China) was used to track the animal.

In addition, as described in our publication (22) spatial working memory performance was assessed a week after antibody infusion, by recording spontaneous alternation performance in a Y-maze. The maze was made of gray-painted vinyl-chloride. Each arm was 50 cm long, 30 cm high, and 10 cm wide and converged at an equal angle. Each rat was placed at the center of the maze and allowed to move freely through it during an 8 min period. The numbers of arm entries were recorded for 8 min. An alternation was defined as entries into all arms. The percentage of alternation was calculated as (actual alternations /total entered-2) × 100.

### Statistical Analysis

All statistical analyses were performed using SPSS (windows version 13.0). Experimental data (amplitude of mEPSCs and % spontaneous alternation performance were analyzed using one-way ANOVA and experimental data of water maze (swimming path length and swimming speed) were analyzed using two-way repeated ANOVA. As appropriate Tukey's *post hoc* analyses were utilized to determine differences between groups. All values were presented as mean ± standard error. Differences were considered significant at *P* < 0.05.

## Results

### Protein Expression of NR1 and GluR2/3 Expression

**Figure 1:**
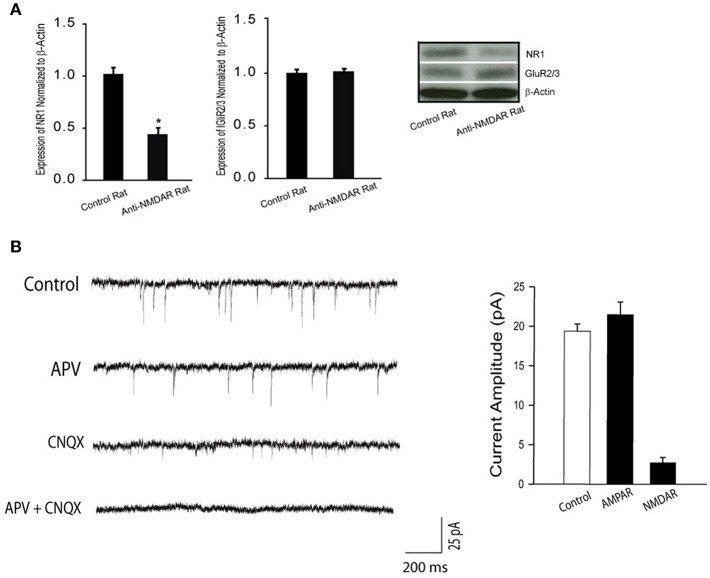
**(A)** Protein expression NR1 and GluR2/3 in the hippocampus of control rats and rats treated with anti-NMDAR. Treatment of anti-NMDAR attenuated the protein levels of NMDA NR1 but not AMPA GluR2/3. ^*^P < 0.05 vs. control rats. The number of rats = 6 in each group. **(B)** Typical traces and averaged data, showing NMDAR- and AMPAR-mediated mEPSCs. The mEPSCs were recorded in perfusion solution with TTX, picrotoxin, and 0 Mg^2+^ to isolate synaptic glutamate-mediated currents. APV, an NMDA receptor antagonist, blocks mEPSCs, allowing AMPAR-mediated currents observed. CNQX, an AMPA receptor antagonist, blocks mEPSCs, allowing NMDAR-mediated currents observed. As APV plus CNQX were applied, mEPSCs were completely blocked. Twelve neurons were used in this experiment. Under the same recording conditions, NMDAR-mediated mEPSCs were examined in the hippocampal neurons of control rats and rats with anti-NMDAR that were presented in Figure 2.

In order to determine the effectiveness of anti-NMDAR after its administration, we examined the protein levels of NMDA receptor NR1 and AMPA receptor GluR2/3 in the hippocampus of control rats (*n* = 6) and rats treated with anti-NMDAR (*n* = 6). Figure 1A shows that NR1 was significantly downregulated after application of anti-NMDAR as compared with control rats (*P* < 0.05, between control and anti-NMDAR). However, anti-NMDAR did not alter the protein levels of GluR2/3 expression in the hippocampus (*P* > 0.05, between two groups).

### Synaptic NMDAR and AMPAR Currents

First, we used whole-cell patch recordings of mEPSCs to assess NMDAR- and AMPAR- mediated currents in the hippocampal neurons (number of neurons = 12) of rats without treatment. Figure 1B demonstrates that the mEPSCs were examined at −70 mV in extracellular solutions containing 0 Mg^2+^. TTX was used to block action potentials; and picrotoxin was used to block GABA receptor-mediated miniature currents. As shown in this figure (typical traces and averaged data), APV and CNQX effectively blocked NMDAR- and AMPAR-mediated currents.

**Figure 2:**
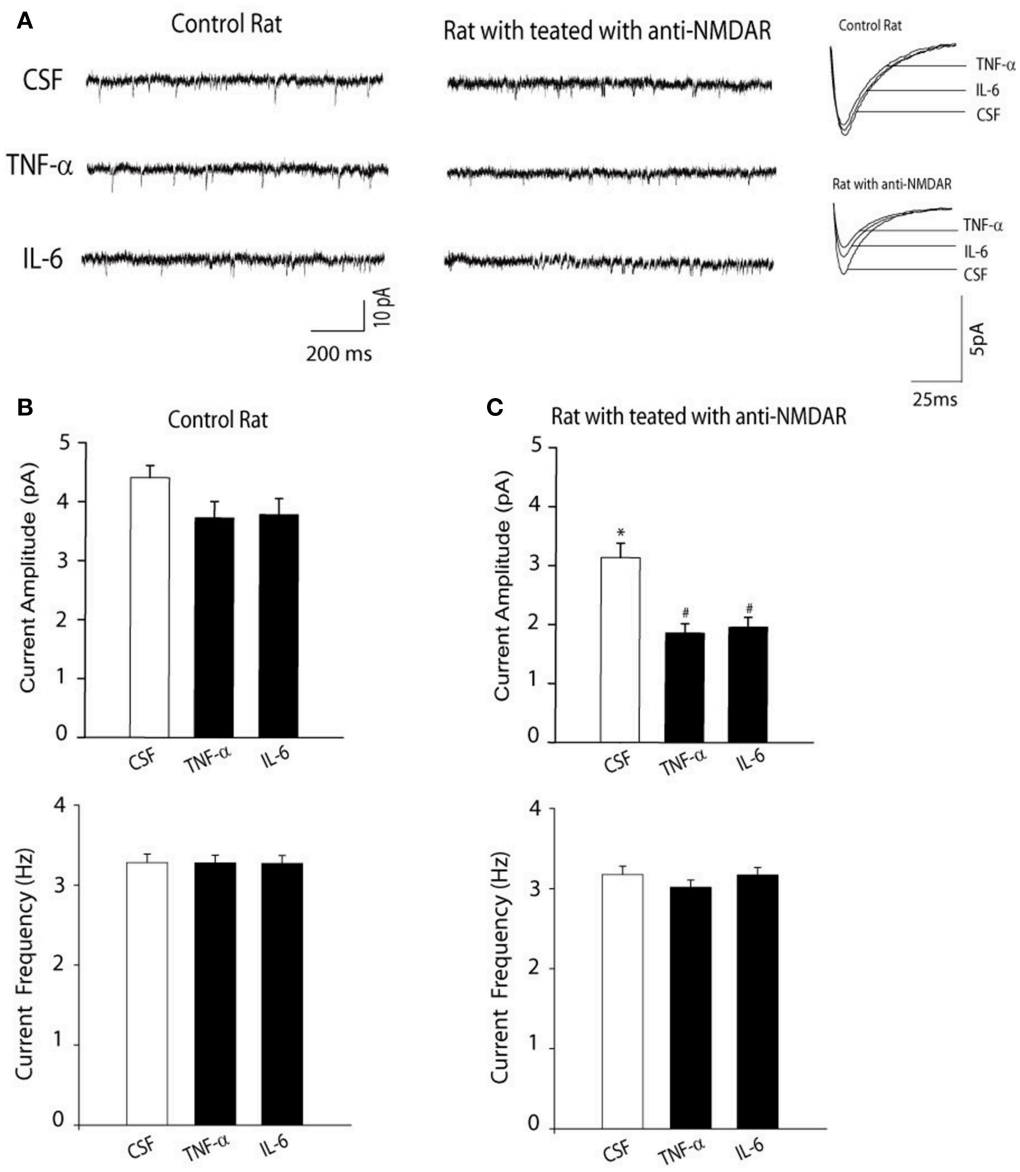
**(A)** Representative traces and **(B,C)** averaged data showing amplitude of NMDAR-mediated mEPSCs was decreased in the hippocampal neurons of rats with anti-NMDAR. The frequency of NMDAR-mediated mEPSCs was not altered. The decreases in NMDAR-mediated mEPSCs became greater in the hippocampal neurons of rats with anti-NMDAR after the prior infusion of TNF-α or IL-6. The effects of TNF-α or IL-6 were observed to be insignificant in control animals. ^*^P < 0.05 vs. control rats with CSF; and ^#^*P* < 0.05, TNF-α/IL-6 vs. CSF in rats with anti-NMDAR. The number of neurons = 10–15 in each group of experiments.

Next, we examined the effects of anti-NMDAR on NMDAR activity using whole-cell patch recordings of mEPSCs. Figure 2 demonstrates that amplitude of NMDAR-mediated mEPSCs in neurons of rats treated with anti-NMDAR (number of neurons = 15) was decreased as compared with the amplitude of NMDAR-mediated mEPSCs in CSF control rats (number of neurons = 10; *P* < 0.05, anti-NMDAR *vs*. control). However, frequency of NMDAR-mediated mEPSCs was not significantly changed by anti-NMDAR. In addition, the prior i.c.v. infusion of TNF-α and IL-6 significantly attenuated amplitude of NMDAR-mediated mEPSCs in neurons of rats treated with anti-NMDAR (number of neurons = 12 in each group; *P* < 0.05, TNF-α/IL-6 vs. CSF control), but no significant alteration was observed in neurons of control rats although TNF-α and IL-6 tended to decrease amplitude of NMDAR-mediated mEPSCs in control rats.

In contrast, anti-NMDAR was observed to have the minimal effects on amplitude of AMPAR-mediated mEPSCs in the hippocampal neurons. i.e., amplitude of AMPAR-mediated mEPSCs was 21 ± 3 pA in the hippocampal neurons of control rats; and 19 ± 3 pA in the hippocampal neurons of rats treated with anti-NMDAR (*P* > 0.05, control vs. anti-NMDAR; number of neurons = 8 in each group).

### Learning Performance

**Figure 3:**
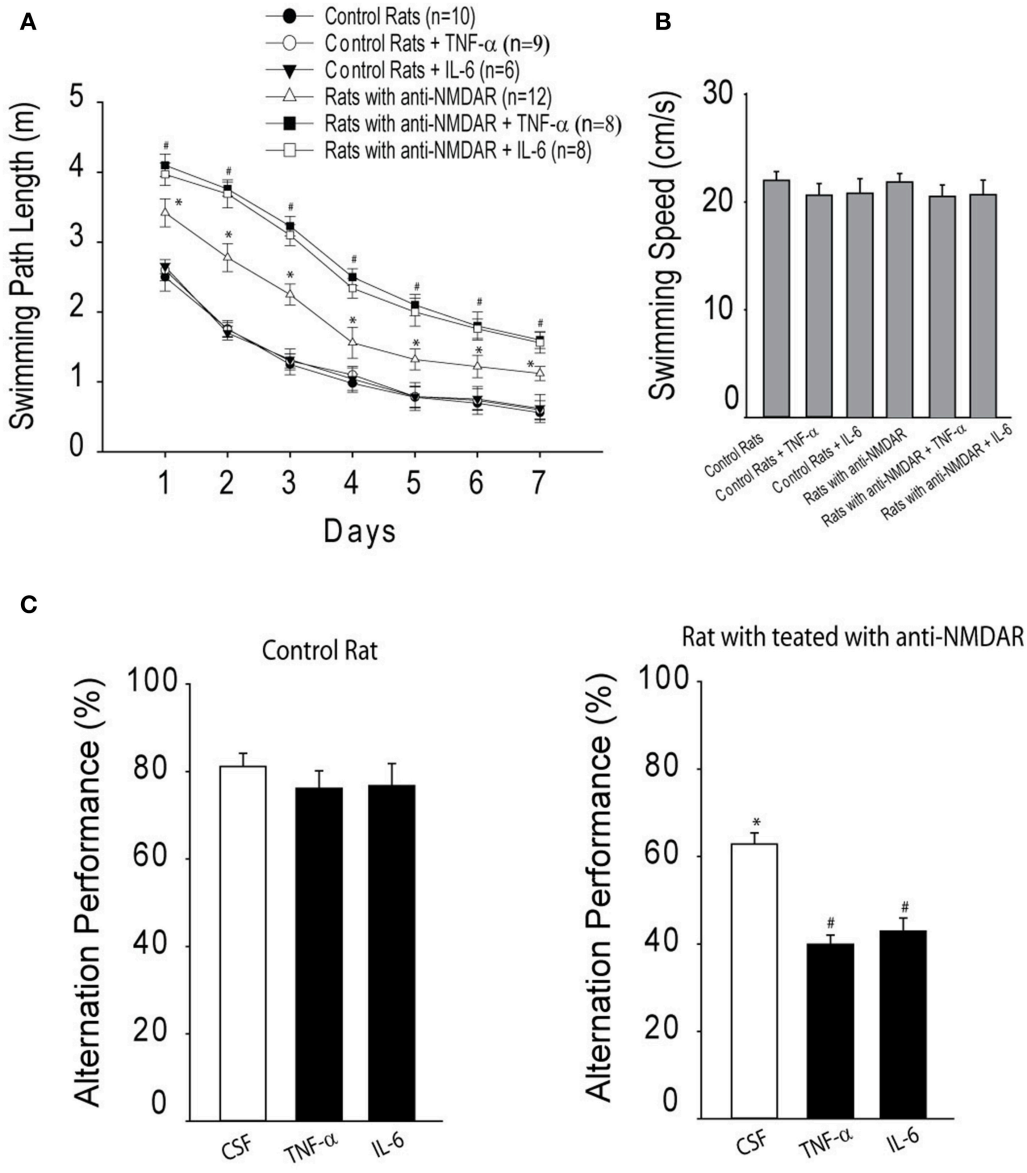
**(A)** The cumulative swimming path length on 7 consecutive days in the Morris water maze for six experimental groups, showing increases of swimming path length in rats that have been injected with anti-NMDAR as compared to control rats. TNF-α or IL-6 amplified increases of swimming path length observed in rats anti-NMDAR, but not in control rats. ^*^P < 0.05 vs. control rats; and ^#^*P* < 0.05, TNF-α/IL-6 vs. CSF control. **(B)** There were insignificant differences observed in the swimming speed in these different experimental groups (*P* > 0.05). **(C)** The effects of anti-NMDAR on spontaneous alternation performance in rats. The learning performance was examined after infusion of TNF-α or IL-6. ^*^P < 0.05, control rats vs. rats treated with anti-NMDAR. ^#^*P* < 0.05, TNF-α/IL-6 vs. CSF control. The number of animals in each group is shown in the figure.

As illustrated in Figure 3A, two-way repeated ANOVA shows that distance to locate the platform was decreased across the six training sessions in three groups of control rats [*F*_(3,32)_ = 19.63; *P* < 0.001]. There were no significant effects by session interaction for decreased distance to find the platform (*P* = 0.75). Likewise, in three groups of rats with anti-NMDAR distance to locate the platform was also decreased across the six training sessions [*F*_(3,32)_ = 17.67; *P* < 0.001]. No significant effects by session interaction was observed for decreased distance to find the platform (*P* = 0.83). In addition, the swimming path length cumulatively for six consecutive trials was greater in rats injected with anti-NMDAR as compared to control animals injected with CSF (*P* < 0.01, anti-NMDAR rats/*n* = 12 vs. control rats/*n* = 10). Note that TNF-α/or IL-6 amplified the swimming path length observed in rats anti-NMDAR (*P* < 0.01, TNF-α/IL-6/*n* = 8 in each group vs. CSF), but the minimal effects of TNF-α or IL-6 were observed in control rats. Also, to assess the possible effects of motor activity in rats we examined swimming speed and Figure 3B shows that there were insignificant differences in the swimming speed in those experimental groups (*P* > 0.05 among groups).

Moreover, spontaneous alternation performance was examined. In this experiment, as a measure of activity level the number of arm entries was determined by counting the number of arms entered in the maze for each animal during the test. Insignificant differences in the number of arm entries were found between control group and anti-NMDAR group. The number of arm entries for each group was 15.8 ± 2.6 in control rats (*n* = 8) and 16.2 ± 2.8 in rats with anti-NMDA (*n* = 10; *P* > 0.05 between two groups). Then, spatial working memory performance was examined. Figure 3C demonstrates that the percentage of spontaneous alternation was decreased in rats with anti-NMDAR (*n* = 12) as compared with control rats (*n* = 10; *P* < 0.05, between control and anti-NMDAR). A decrease of spontaneous alternation was greater in rats with anti-NMDAR after infusion of TNF-α or IL-6 (*n* = 8 in each group; *P* < 0.05, TNF-α/IL-6 vs. CSF). However, TNF-α/or IL-6 had insignificant effects on spontaneous alternation in control animals (*P* > 0.05, TNF-α/IL-6 vs. CSF).

## Discussion

Prior studies have shown that NMDAR antibody pathogenicity leads to neuronal surface receptor downregulation, subsequent impairment of NMDAR-mediated currents and behavior abnormalities (24, 25). Consistent with those findings, data of our current study specifically demonstrated that NMDAR-mediated mEPSCs in the hippocampal neurons of rats treated with anti-NMDAR were attenuated; and anti-NMDAR impaired learning performance in rats. In addition, after CSF containing anti-NMDAR was stereotactically injected into the dentate gyri of rats (16), substantial deficits in NMDAR-mediated synaptic transmission and plasticity were observed later *in vitro* after *in vivo* application of anti-NMDAR. In addition, in this prior study (16), Morris water maze experiments showed impairments in learning behavior in the rats injected with anti-NMDAR since the swimming path length cumulatively for consecutive trials was observed to be greater in rats injected with anti-NMDAR as compared to control animals injected with CSF. In the similar way, we injected anti-NMDAR antibody into the dentate gyri of rats in the current study and we found that consistent results demonstrating increases of the swimming path length in rats with anti-NMDAR. Our current results involved additional indication that the prior cerebral infusion TNF-α and IL-6 amplified the decreases of NMDAR-mediated mEPSCs in the hippocampal neurons with anti-NMDAR and this worsened learning performance. This result also provides potential evidence that TNF-α and IL-6 are engaged in the abnormalities in NMDAR-mediated mEPSCs and learning performance in anti-NMDAR encephalitis.

In patients with anti-NMDAR encephalitis, the autoantibodies are first found in the serum and CSF and then high antibody concentrations are seen in intrathecal fluid (3, 26). In general, functional NMDAR has two NR1 and two NR2 subunits, but anti-NMDAR is localized to the N-terminal extracellular loop of the NMDAR subunit NR1 (27). The N-terminal extracellular domain of NR1 is recognized in all patients' antibodies in studying an antibody-mediated pathogenesis (3). In the disease, the extracellular domain of the NR1 subunit of the NMDAR is directly targeted by autoantibodies (3, 4). Also, noticeable psychiatric and behavioral symptoms, rapid memory loss, seizures, abnormal movements, hypoventilation, and autonomic instability are presented in those patients (3, 4, 28). A study using *in vitro* and *in vivo* methods has further indicated the cellular mechanisms by which patients' antibodies result in a decrease in NMDAR density and function in cell surface and synaptic site (14). This is likely to lead to the learning, memory, and other behavioral deficits observed in patients with anti-NMDAR encephalitis. Nonetheless, the underlying mechanisms leading to antibody-mediated dysfunction in this disease are largely unknown.

Interestingly, a study showed that injection of patient's CSF into the rat hippocampus led to an NMDAR phenotype similar to key clinical features such as memory disturbance (16). In this prior study, the same results were found after anti-NMDAR1 was injected into the dentate gyrus of rats. Thus, in our current report, anti-NMDAR1 was given into the hippocampus of rats and we found that the protein levels of NR1 were downregulated in the hippocampus after injection of anti-NMDAR, suggesting its effectiveness on attenuating NMDAR. In addition, we found that amplitude of NMDAR-mediated mEPSCs was decreased after injection of anti-NMDAR into the hippocampus. Of note, expression of AMPA GluR2/3 and AMPAR-mediated mEPSCs were not affected by treatment of anti-NMDAR. It should be noted that less expression of NR1 was observed in the current study it was likely that part of the NR1 subunits were already bound to the antibody administered.

Synaptic plasticity plays a role in regulating memory, learning, and cognition (5, 13). The proper synaptic localization and trafficking of the excitatory glutamate NMDA and AMPA receptors are necessary to modulate synaptic plasticity and these neurological functions (6). Using animal models the glutamate receptors are genetically or pharmacologically altered and the roles played by these receptors at the synaptic and cellular levels have been documented (29). In contrast, in human studies, indirect approaches are used to determine the role of these receptors in memory, learning, cognition and psychosis. For example, pharmacological trials (e.g., psychosis of NMDAR antagonists) (30) and analysis of brain tissue from patients with Alzheimer's disease or schizophrenia reveal several molecular pathways causing a downstream alteration of glutamate receptors (31).

Nonetheless, in animal studies, the Morris water maze hidden platform task was used to assess space learning, which is considered as a hippocampus-specific learning paradigm and depends on NMDAR activation (32). Considering that impaired spatial working memory was observed after granule cell-specific disruption of the NR1 gene (33), we further examined whether this behavioral task was likely affected in rats injected with anti-NMDAR. Consistent with our data showing the decreased amplitude of NMDAR-mediated mEPSCs of anti-NMDAR rats, learning performance was impaired in these animals. In this experiment, we did not find any significant differences in the swimming speed in control rats and rats with anti-NMDAR, suggesting that motor activity has the minimal effects on learning performance in rats treated with anti-NMDAR.

Evidence has demonstrated that PICs (i.e., TNF-α) are engaged in memory function and synaptic plasticity in the hippocampus (34). Under physiological conditions, TNF-α can increase AMPA receptors into the cell membrane and this process is important for synaptic scaling (35). Synaptic scaling is a form of synaptic plasticity alleviating the neuronal excitability by adjusting the strength of all of the excitatory synapses of an individual neuron (36). However, at pathological concentration, TNF-α is injurious to memory and synaptic plasticity. For example, up-regulation of TNF-α is associated with deficits of memory and synaptic plasticity in Alzheimer's disease, and inhibition of TNF-α is effective for treating the disease (37, 38). Nevertheless, the mechanisms by which PICs impair synaptic plasticity and memory are mostly unknown in anti-NMDAR encephalitis although the studies have shown that some cytokines are elevated in CSF of patients with anti-NMDAR encephalitis and, in general, neuroinflammation contributes to the severity of anti-NMDAR encephalitis (17, 18). In the present work, we showed that a chronic infusion of TNF-α into the central nervous system decreased NMDAR-mediated EPSCs in the hippocampal neurons, suggesting that amplification of TNF-α likely worsens dysfunction of NMDARs in anti-NMDAR encephalitis. Indeed, we found that chronic application of TNF-α amplified impairment of the learning performance in animals treated with anti-NMDAR.

IL-6 is an immune cell mediator in the periphery in involvement of the modulation of neurological functions (39). Under normal conditions, IL-6 expression is low in the brain, but it increases largely in neurological diseases such as stroke, brain damage and seizures (40, 41). Due to neuroinflammation, an increase in the levels of endogenous IL-6 in the brain contributes to pathogenesis of some neurodegenerative diseases (42, 43). A prior study has also shown that IL-6 decreases NMDA-induced cytosolic Ca^2+^ overload thereby inhibiting neuronal apoptosis and necrosis, suggesting a neuroprotection of IL-6 (44). Nonetheless, in our current study, a chronic infusion of IL-6 attenuated NMDAR-mediated mEPSCs in the hippocampal neurons of rats treated with anti-NMDAR and this worsened learning performance in animals. Thus, IL-6 may exert a bidirectional effect on synaptic plasticity and memory. Normalizing IL-6 production and its levels is likely a better strategy to treat numerous neurological disorders observed in anti-NMDAR encephalitis.

There are possible synaptic mechanisms by which cytokines can worsen the effects of anti-NMDAR on NMDA receptor currents. In a prior study, it was observed that TNF-α enhanced the frequency of spontaneous EPSCs, whereas IL-6 reduced the frequency of spontaneous IPSCs in neurons of isolated spinal cord slices (45). In contrast, IL-1β enhanced the frequency and amplitude of sEPSCs and reduced the frequency and amplitude of sIPSCs. In addition, TNF-α and IL-1β enhanced AMPA- or NMDA-induced currents, and IL-1β and IL-6 suppressed GABA- and glycine-induced currents (45). Those findings suggest that PICs induce central sensitization via distinct and overlapping synaptic mechanisms in superficial dorsal horn neurons either by increasing excitatory synaptic transmission or by decreasing inhibitory synaptic transmission. It is assumed that anti-NMDAR is likely to decrease central sensitization to a greater degree in synaptic sites of the hippocampal neurons via such synaptic mechanisms and application of TNF-α and IL-6 worsens its effects.

### Study Limitations

A prior study demonstrated that the inhibition of TNF-α synthesis can significantly reverse hippocampus-dependent cognitive deficits induced by chronic neuroinflammation (46), suggesting that TNF-α is a critical mediator of chronic neuroinflammation-induced neuronal dysfunction and cognitive impairment. Our results showed that TNF-α and IL-6 are engaged in NMDAR-mediated currents and behavior abnormalities. However, it is needed in the further study examining the prevention of the observed effect of NMDAR antibody on the amplitude and amount of NMDA-receptors as well as cognitive functions after application of blockers of TNF-α and IL-6. In addition, a few issues need to be acknowledged. i.e., histological experiments designed to show the interaction of the antibody and NMDA-receptor and additional experiments for evaluation of LTP in rat hippocampus to show the electrophysiological correlation with learning disturbance.

In conclusion, our findings suggest that neuroinflammation plays a role in attenuating NMDAR-mediated mEPSCs and exacerbating the memory impairment observed in rats with anti-NMDAR encephalitis. Anti-inflammation should be considered in improving the memory impairment in anti-NMDA encephalitis.

## Ethics Statement

All animal protocols were in accordance with the guidelines of the International Association for the Study of Pain and approved by the Institutional Animal Care and Use Committee of Jilin University.

## Author Contributions

XW and CM designed the studies, collected experimental and analyzed data, and drafted the paper. C-YL and G-JL have participated in performing experiments and assist in data analysis. DZ and D-FH designed the studies and participated in data analysis and reviewed the draft.

### Conflict of Interest Statement

The authors declare that the research was conducted in the absence of any commercial or financial relationships that could be construed as a potential conflict of interest.
